# Efficacy of furosemide for treatment of liver cirrhosis: A systematic review protocol of randomized controlled trial

**DOI:** 10.1097/MD.0000000000015300

**Published:** 2019-04-19

**Authors:** Zheng-Ri Zhu, Wan-Lu Liu, Zhao-Min Ding, Yue Li

**Affiliations:** a Second Ward of Gastroenterology Department; b Department of Nursing Care; c Department of Cardiac Intensive Care Medicine; d Department of Nephrology, The Affiliated Hongqi Hospital of Mudanjiang Medical University, Mudanjiang, China.

**Keywords:** efficacy, furosemide, LC, randomized controlled trial, safety, systematic review

## Abstract

**Background:**

Previous clinical studies have reported that furosemide can be used to treat liver cirrhosis (LC) effectively. However, no study systematically explored this issue. This systematic review aims to investigate the efficacy and safety of furosemide for treatment of LC.

**Methods:**

This study will be conducted through searching the following literature sources from their inception to February 28, 2019 without any language limitations: PUBMED, EMBASE, PsycINFO, Web of Science, Scopus, OpenGrey, Cochrane Library, Cumulative Index to Nursing and Allied Health Literature, Allied and Complementary Medicine Database, and Chinese Biomedical Literature Database. In addition, reference lists of relevant reviews and websites of clinical trial registry will also be searched. Only randomized controlled trials of furosemide for treatment of LC will be included in this study. Two reviewers will independently select studies, collect data, and determine risk of bias. RevMan 5.3 software will be used to pool the data and to conduct meta-analysis if sufficient studies will be included with acceptable heterogeneity.

**Results:**

This study will investigate the efficacy and safety of furosemide for LC by the assessment of primary and secondary outcomes. The primary outcome includes mortality rate. The secondary outcomes consist of response rate, overall survival, body weight, urinary volume, quality of life, as measured by any relevant scales, and adverse events.

**Conclusion:**

The results of this study may provide summarized evidence of furosemide for the treatment of LC.

**Ethics and dissemination:**

No individual patient data will be used in this study, thus no ethics approval is needed. The findings of this study will be published in peer-reviewed journals.

## 1 Introduction

Liver cirrhosis (LC) is a very common life-threatening hepatic disorder in patients with hepatic diseases.^[1–3]^ Many factors are responsible for this disorder, such as adequate alcohol consumption, viral hepatitis B and C, metabolic abnormal condition, as well as the non-alcoholic fatty liver disease.^[4–8]^ Clinical manifestations often present as ascites, jaundice, gastrointestinal hemorrhage, and hepatic encephalopathy, which dramatically impair the quality of life in patients with LC.^[9–11]^ It is reported that more than 1 million people died of LC, which accounts for almost 2% of global deaths.^[12–13]^

Furosemide, a loop diuretic, is used for inhibiting chloride and sodium reabsorption in the thick ascending limb of the loop of Henle.^[14]^ It can rapidly absorb from gut and its action can reach peak effect quickly within 1 to 2 hours, and then diuretic effects end in 3 to 4 hours after consumption.^[15]^ Many studies have reported that furosemide is widely utilized for the treatment of patients with LC.^[16–22]^ However, up to date, no systematic study has been conducted to investigate the efficacy and safety of furosemide for patients with LC. Thus, this study will explore the efficacy and safety of furosemide for LC systematically.

## 2 Methods

### 2.1 Study registration

This study protocol has been registered at PROSPERO (CRD42019126000). It has been reported following the guidelines of Preferred Reporting Items for Systematic Reviews and Meta-Analysis Protocol statement.

### 2.2 Eligibility criteria

#### 2.2.1 Study types

This study will only consider randomized controlled trials (RCTs) of furosemide for the treatment of LC. Any other types will be excluded, such as animal studies, reviews, comments, letters, expert options, case studies, observational studies, non-controlled studies, and non-RCTs.

#### 2.2.2 Intervention types

The experimental treatment includes any forms of furosemide monotherapy. However, the combination of furosemide with other treatments will be excluded. The control intervention can be any therapies, but not the furosemide.

#### 2.2.3 Participant types

Patients with clinically diagnosed with LC will be included, regardless of their race, sex, and age.

#### 2.2.4 Outcome measurement types

Primary outcome is mortality rate. Secondary outcomes are response rate, overall survival, body weight, urinary volume, quality of life, as measured by any relevant scales, and adverse events.

### 2.3 Search strategy

We will search the following literature sources from their inception to February 28, 2019 without any language limitations: PUBMED, EMBASE, PsycINFO, Web of Science, Scopus, OpenGrey, Cochrane Library, Cumulative Index to Nursing and Allied Health Literature, Allied and Complementary Medicine Database, and Chinese Biomedical Literature Database. Additionally, we will also search reference lists of relevant reviews and clinical trial registry. All RCTs of furosemide for treatment of LC will be considered in this study. We will provide search strategy sample of Cochrane Library and will be shown in Table 1. Similar search strategy details will be applied to other literature sources.

**Table 1:**
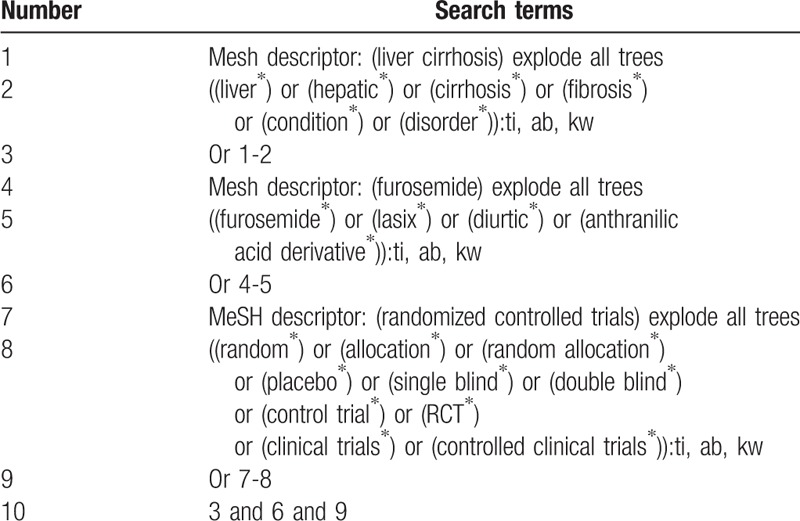
Search strategy for database of Cochrane Library.

### 2.4 Study selection

The entire study selection will be performed by 2 independent reviewers. After removing duplicated studies, all records will be reviewed through titles and abstracts, and unqualified studies will be ruled out. The full text of the remaining studies will be further assessed for inclusion. A third reviewer will be invited to solve any disagreements arise between 2 reviewers through discussion. We will record the reasons of exclusions for each ruled out studies. The whole process of study selection will be followed and presented in the flowchart of Preferred Reporting Items for Systematic Reviews and Meta-Analysis Protocol statement.

### 2.5 Data collection and management

Data collection from each included study will be carried out independently by 2 reviewers. Any opposition will be resolved by consensus with a third independent reviewer. The extracted information will comprise of first author, published year, region, setting, participant characteristics (race, age, etc), sample size for both experimental and control groups, exclusion and inclusion criteria, intervention details in both experimental and control groups, and outcome measurements (primary, secondary, and safety).

### 2.6 Missing data dealing with

Any insufficient information will be contacted the primary authors to require those data. If those data are not achievable, we will only analyze the available data and will discuss it as a limitation.

### 2.7 Risk of bias

The methodological quality for each eligible study will be assessed by using Cochrane risk of bias tool. It will be evaluated by through 7 domains, and each domain will be assessed as high risk of bias, unclear risk of bias, or low risk of bias. Two reviewers will independently assess the risk of bias. In case of any disagreements, a third reviewer will be consulted to settle down those divergences.

### 2.8 Treatment effect measurement

In this study, standardized mean difference and 95% confidence intervals (CIs) will be used for continuous data, while risk ratio and 95% CIs will be utilized for dichotomous data.

### 2.9 Heterogeneity assessment

To test the heterogeneity among the included studies, I^2^ test will be used for the identification. Reasonable heterogeneity will be considered if I^2^ ≤50%. On the other hand, substantial heterogeneity will be considered if I^2^ >50%.

### 2.10 Data synthesis

If reasonable heterogeneity will be identified, a fixed-effect model will be used to pool the data. In addition, meta-analysis will be conducted. If substantial heterogeneity will be detected, a random-effect model will be used to pool the data. In such case, subgroup analysis will be carried out to identify any potential factors that may cause the substantial heterogeneity. If the considerable heterogeneity still exists after subgroup analysis, the data will not be pooled, and meta-analysis will also not be conducted. However, a narrative summary will be reported.

### 2.11 Subgroup analysis

Subgroup analysis will be carried out based on the different interventions of experimental and control groups, or outcome measurement instruments.

### 2.12 Sensitivity analysis

Sensitivity analysis will be performed to check the robustness of synthesized results by removing low-quality studies.

### 2.13 Reporting bias

Funnel plot and Egger regression test will be used to check any possible publication bias if sufficient studies are included.

## 3 Discussion

Previous studies have hypothesized that furosemide has been used for the treatment of patients with LC.^[16–22]^ However, all those studies have been conceptual. Considering a variety of clinical trials on assessing the efficacy of furosemide for LC, the present study aims to perform a systematic research synthesis to inform the efficacy and safety of furosemide for LC. We wish to summarize the most up-to-date evidence of furosemide treatment of LC. The findings of the present study are expected to inform either researchers or patients and clinicians focusing on the public health approach to education.

## Author contributions

**Conceptualization:** Zheng-Ri Zhu, Wan-Lu Liu, Zhao-Min Ding, Yue Li.

**Data curation:** Zheng-Ri Zhu, Wan-Lu Liu, Yue Li.

**Formal analysis:** Zheng-Ri Zhu, Wan-Lu Liu, Zhao-Min Ding.

**Funding acquisition:** Zheng-Ri Zhu.

**Investigation:** Yue Li.

**Methodology:** Wan-Lu Liu, Zhao-Min Ding.

**Project administration:** Yue Li.

**Resources:** Zheng-Ri Zhu, Wan-Lu Liu, Zhao-Min Ding.

**Software:** Zheng-Ri Zhu, Wan-Lu Liu, Zhao-Min Ding.

**Supervision:** Yue Li.

**Validation:** Zheng-Ri Zhu, Wan-Lu Liu, Zhao-Min Ding, Yue Li.

**Visualization:** Zheng-Ri Zhu, Yue Li.

**Writing – original draft:** Zheng-Ri Zhu, Yue Li.

**Writing – review & editing:** Zheng-Ri Zhu, Wan-Lu Liu, Zhao-Min Ding, Yue Li.
